# Interfacial Spin Glass State and Exchange Bias in the Epitaxial La_0.7_Sr_0.3_MnO_3_/LaNiO_3_ Bilayer

**DOI:** 10.1186/s11671-017-2110-0

**Published:** 2017-05-04

**Authors:** Guo-wei Zhou, Xiao-fen Guan, Yu-hao Bai, Zhi-yong Quan, Feng-xian Jiang, Xiao-hong Xu

**Affiliations:** 1 School of Chemistry and Materials Science, Key Laboratory of Magnetic Molecules and Magnetic Information Materials, Ministry of Education, Shanxi Normal University, Linfen, 041004 People’s Republic of China; 2 Research Institute of Materials Science, Shanxi Normal University, Linfen, 041004 China; 3 School of Physics and Electronic Information, Shanxi Normal University, Linfen, 041004 People’s Republic of China

**Keywords:** Magnetic materials, Interfaces, Stack order, Spin glass state, Exchange bias

## Abstract

We study the magnetic properties of an epitaxial growth bilayer composed of ferromagnetic La_0.7_Sr_0.3_MnO_3_ (LSMO) and paramagnetic LaNiO_3_ (LNO) on SrTiO_3_ (STO) substrates. We find that the stack order of the bilayer heterostructure plays a key role in the interfacial coupling strength, and the coupling at the LSMO(top)/LNO(bottom) interface is much stronger than that at the LNO(top)/LSMO(bottom). Moreover, a strong spin glass state has been observed at the LSMO/LNO interface, which is further confirmed by two facts: first, that the dependence of the irreversible temperature on the cooling magnetic field follows the Almeida-Thouless line and, second, that the relaxation of the thermal remnant magnetization can be fitted by a stretched exponential function. Interestingly, we also find an exchange bias effect at the LSMO/LNO bilayer below the spin glass freezing temperature, indicating that the exchange bias is strongly correlated with the spin glass state at its interface.

## Background

With the rapid progress of modern growth techniques, the development of high quality artificial heterostructures could lead to the discovery of unexpected physical properties and emergent functionalities, such as orbital reconstruction, exchange bias, interface superconductivity, and magnetoelectric coupling [1–4]. The discovery of the exchange bias (EB) effect by Meiklejohn and Bean is fascinating for its many potential applications in spin valves, magnetic recording, and magnetic read heads, among other things [5–9]. The exchange bias as the interfacial phenomenon in this system has prompted several decades of experimental and theoretical work in the heterostructures of ferromagnetic (FM) and antiferromagnetic (AFM) materials [10–13]. Interestingly, the exchange bias has been observed in the heterostructure interface composed by the ferromagnetic half-metal LaSrMnO_3_ and the paramagnetic (PM) metal LaNiO_3_ [14, 15]. For example, Sánchez et al. observed the unexpected exchange bias effect in the FM/PM bilayer of La_0.75_Sr_0.25_MnO_3_/LaNiO_3_ and explained this phenomenon through the existence of magnetic behavior in the Ni^2+^ and Mn^4+^ by charge transfer [14]. Peng et al. prepared a La_0.7_Sr_0.3_MnO_3_/LaNiO_3_ bilayer in which the LaNiO_3_ is the top layer, and attributed the exchange bias to magnetization frustration induced by orbital reconstruction and charge transfer [15]. In these relatively thin bilayers, the interfacial properties are usually influenced by charge and orbital degrees of freedom, which can be explored by X-ray absorption spectra (XAS). However, for the thicker bilayer, it is difficult to explore the interfacial charge states by XAS due to its shallow (several nanometers) exploring depth. According to Ding et al. and Hyun et al., in the thicker bilayer, the coercivity enhancement and exchange coupling appearance are due to the interfacial spin glass state and magnetic structure changes rather than charge transfer [16, 17]. Whether the interfacial charge transfer is enough to lead to magnetic coupling in the thinner heterostructure is still a controversial issue. For example, in La_0.7_Sr_0.3_MnO_3_/SrRuO_3_ (FM) superlattices, interfacial magnetic coupling was not primarily controlled by charge transfer [18]. Therefore, the magnetic characteristics of heterostructure interface remain an open question.

In this paper, we report the experimental results of the relatively thick ferromagnetic half-metal La_0.7_Sr_0.3_MnO_3_ and paramagnetic metal LaNiO_3_ bilayer with a width of dozens of nanometers. As reported in previous works, LNO is the only member of the perovskite nickelates family lacking any magnetic order in its bulk form [19, 20]. Through magnetic measurement, we confirm that the LNO layer could not contribute to total magnetization but is the necessary material to produce the interfacial coupling. First, we explore the influence of the deposition sequence of LSMO and LNO layers on the intensity of the interfacial coupling. Next, we find that the stronger interfacial coupling in the LSMO/LNO bilayer caused by the spin glass state results in a large enhancement of the coercivity and a clear exchange bias effect.

## Methods

To obtain high quality epitaxial films, all the samples were grown on an atomically flat TiO_2_-terminated SrTiO_3_ (100) substrate which was set-etched with buffered HF acid. The samples were deposited by pulsed laser deposition (PLD) that could be monitored in situ, assisted with reflection high-energy electron diffraction (RHEED). The deposition was done using a 248-nm KrF excimer laser at a temperature of 725 °C, and oxygen pressure of 100 mTorr. After the growth, the samples were in situ annealing for 1 h in an oxygen pressure 300 Torr. In this work, we prepared four different types of samples using 25-nm LSMO and 35-nm LNO single layer films, a LNO (35 nm)/LSMO (25 nm) bilayer where LNO is the top layer and a LSMO (25 nm)/LNO (15, 25, 35 nm) bilayer reversing the deposited sequence. The structure quality and orientation of the samples were analyzed by X-ray diffraction (XRD) using Cu Kα radiation. The surface morphology of the substrate was measured by atomic force microscopy (AFM). The magnetic properties of the samples were measured using vibrating sample magnetism (PPMS-VSM) and an applied magnetic field parallel to the sample plane.

## Results and Discussion

**Fig. 1:**
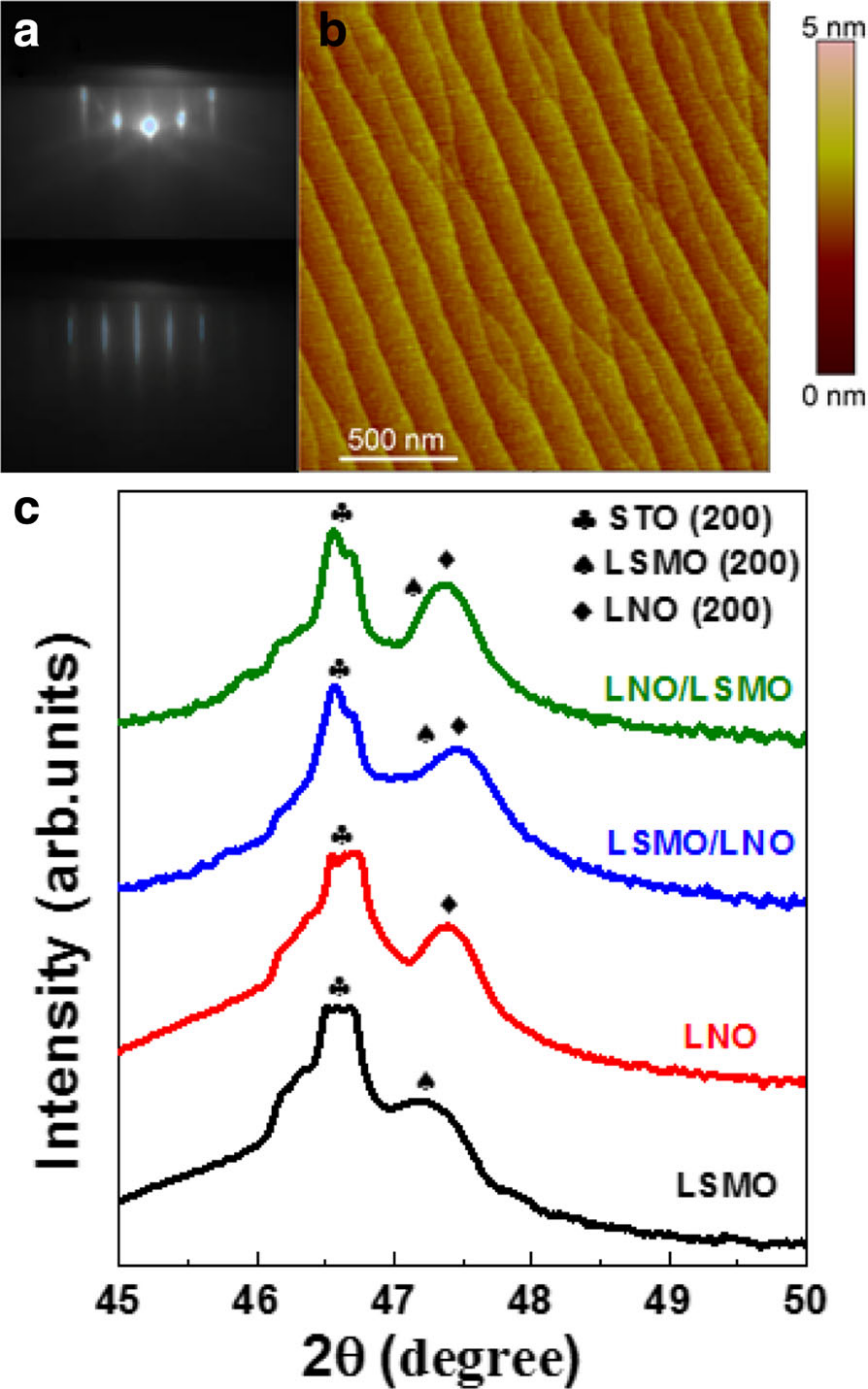
**a** RHEED patterns of the STO substrate before and after the growth of the LSMO/LNO bilayer; **b** AFM topography image (2 × 2 μm^2^) of the STO substrate. The step height corresponds to one monolayer; **c** XRD spectra of all four types of samples grown on the STO substrate

All the samples exhibit epitaxial growth, for instance, the RHEED patterns of the STO substrate and the LSMO/LNO bilayer at the end of growth are shown in Fig. 1a. The streak pattern with the Laue circles and the strongly developed Kikuchi lines clearly exclude the possibility of faceted morphology in the bare STO (100) substrate. At the same time, the direct AFM image of the substrate in Fig. 1b confirms an atomically flat resulting surface that exhibits step-and-terrace morphology and an average surface roughness of less than 0.246 nm. We also observe clear Kikuchi lines in the RHEED pattern after the growth process of the bilayer, which confirm that a high quality sample is obtained in the layer-by-layer model. In order to further survey structural quality and orientation, XRD spectra of the four samples, LSMO and LNO single layers, and LSMO/LNO and LNO/LSMO bilayers are measured using Cu Kα radiation in Fig. 1c. The results suggest that the samples possess high quality crystallinity and *c*-axis orientation properties. In the LNO/LSMO and LSMO/LNO bilayers, the diffraction peaks of the LSMO layer are inconspicuous and may be overlapped with those of the substrates and the LNO layers. The perpendicular *c*-axis lattice parameters of the LNO and LSMO single layers are calculated as 3.841 and 3.865 Å respectively, which is smaller than their bulk values. Thus, both the LNO and LSMO layers deposited on STO substrates sustain an in-plane tensile strain. It is obvious that the peaks of LNO in bilayers have a slight shift to the right compared with those in the corresponding single layer, which is caused by the additional tensile strain by the LSMO layer.

**Fig. 2:**
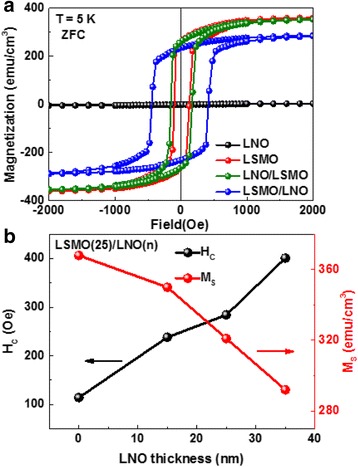
**a** Magnetic hysteresis loops of the four different samples at 5 K after zero field-cooling from room temperature. **b** The *H*_C_ and *M*_S_ of LSMO/LNO bilayer dependence on the LNO layer having different thickness. The *M*_S_ was obtained after saturating at 2 kOe

The hysteresis loops of the four samples measured at 5 K are shown in Fig. 2a. It is obvious that the LNO single layer is typically paramagnetic and could not contribute any magnetic moments, whereas the LSMO single layer is ferromagnetic with a saturation magnetization (*M*_S_) of 360 emu/cm^3^. The coercivity of the LSMO single layer is 115 Oe. The coercivity slightly increases to 160 Oe for the LNO/LSMO bilayer with LNO on the top, and increases dramatically to 401 Oe for the LSMO/LNO bilayer with LNO at the bottom. Moreover, the saturation magnetization of the LNO/LSMO bilayer is almost same as the LSMO single layer and clearly decreases for the LSMO/LNO bilayer. This indicates that the stack order of bilayer heterostructure plays a significant role in the interfacial coupling strength. However, Peng et al. report that the LNO/LSMO bilayer with LNO on the top exhibits strong interfacial coupling, which is not obvious in our experiment [15]. As with previous studies, the charge transfer is considered to be a main factor in determining the interface coupling in the thinner LSMO and LNO heterostructure [14], which often happens on a length scale of a nanometer. However, for the thicker bilayer heterostructure, Ding et al. report that, if the coupling enhancement is due to the charge transfer, it should be independent of the non-magnetic layer thickness [16]. In our case, we fix the LSMO thickness at 25 nm and vary the LNO thickness from 15 to 35 nm. It is obvious that the *H*_C_ increases and the *M*_S_ decreases as the LNO layer thickness increases, as shown in Fig. 2b, which does not follow the mechanism of charge transfer.

**Fig. 3:**
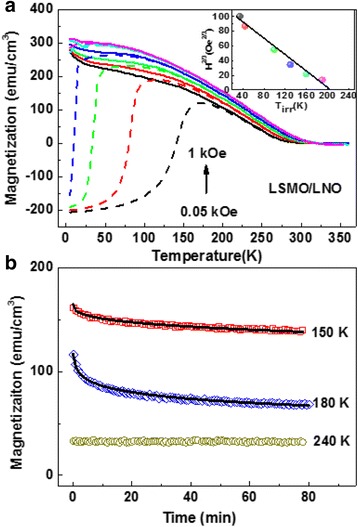
**a** *M*–*T* curves of the LSMO/LNO bilayer heterostructure are measured under different external magnetic fields, *H* = 0.05 (*black*), 0.1 (*red*), 0.2 (*green*), 0.4 (*blue*), 0.8 (*light blue*), and 1 (*purple*) kOe, respectively. The *solid* and *dashed lines* are the FC and ZFC curves. The corresponding plots of *H* ^2/3^ versus *T*_irr_ and linear fitting to Eq. (1) are shown in the inset. **b** Time dependence of the thermal remnant magnetization under the cooled magnetic field of 0.1 kOe and linear fitting to Eq. (2)

In order to clarify the origination of the strong coupling resulting from the increase of *H*_C_ and decrease of *M*_S_, we measure the temperature dependent magnetization (*M*–*T*) curves from 0.05 to 1 kOe for the bilayer of LSMO (25 nm)/LNO (35 nm) with an in-plane magnetic field, shown in Fig. 3a. The magnetization of field-cooling (FC) decreases as the temperature increases and the zero field-cooling (ZFC) increases gradually to the maximum value (*T*_P_) before reducing monotonically. The negative magnetization in the ZFC curves measured in low applied fields may originate from an intrinsic effect of uncompensated spins [21]. The irreversibility temperature (*T*_irr_) also appears to be a bifurcation between the ZFC and FC curves. Based on these phenomena, we analyze the result usually observed for the several commonly known magnetic systems, such as spin glass [22, 23], spin clusters [24, 25], and superparamagnets [26]. Here, *T*_P_ and *T*_irr_ are very close to each other for all applied magnetic fields, and *T*_P_ shifts to low temperatures quickly as the measurement field increases. This is characteristic of the spin glass state and indicates that the spin glass is suppressed by a strong magnetic field [22]. According to the mean-field theory of spin glass, the dependence of *T*_irr_ on field cooling should follow the Almeida-Thouless (AT) line [27, 28]:

1 $$ H\left({T}_{\mathrm{irr}}\right)/\varDelta J\propto {\left(1-{T}_{\mathrm{irr}}/{T}_{\mathrm{F}}\right)}^{3/2}, $$

where the parameter Δ*J* is the width of the distribution of the exchange interaction and *T*_F_ is the zero field spin glass freezing temperature. The linear fit to the experimental data is shown in the inset of Fig. 3a. The fit supports the existence of spin glass behavior in the LSMO/LNO bilayer, and the extrapolation of the AT line gives the spin glass freezing temperature *T*_F_ as 203 K. As Ding et al. reported, the spin glass state in the (FM) LSMO and (G-AFM) SrMnO_3_ bilayer exhibits relaxation of the thermal remnant magnetization (RTRM) below the spin glass freezing temperature [29]. Accordingly, we also measured RTRM curves of the LSMO/LNO bilayer under the cooled magnetic field of 0.1 kOe. Here, we choose three typical temperatures: 150, 180, and 240 K, which are below, around, above the *T*_F_, and apply the stretched exponential function to fit the decay curves at different temperatures:

2 $$ M(t) = {M}_0 \exp \left[- C{\left(\omega t\right)}^{1- n}/\left(1- n\right)\right], $$

where the parameter *ω* is the relaxation frequency, 8.5 × 10^−5^ s^−1^, and *C* is the exponential factor, 0.34 [30]. In Fig. 3b, the fitting parameter *n* is determined to be 0.826 at 180 K and 0.656 at 150 K, which is similar to the values of LSMO/SMO [29]. As the thermal remnant magnetization is quite small at 240 K, which is higher than *T*_F_, the relaxation characterization is not observed. From these results, we suggest that the magnetic relaxation and glassy behavior are most prominent near the freezing temperature of *T*_F_ in the LSMO/LNO bilayer.

The spin glass state in the LSMO/LNO bilayer can be linked to the competition at the interfacial magnetic moment. Because the tensile stress can be existent in both LNO and LSMO layers when they are grown on the STO substrate, the *e*_g_ orbits of the Mn and Ni ions should occupy the *x* ^2^ *–y* ^2^ in LSMO/LNO and LNO/LSMO bilayers [31]. Nevertheless, compared with the LNO/LSMO bilayer, in the case of the LSMO/LNO bilayer, the upper LSMO is compressed by the bottom LNO layer, which induces that the interfacial Mn ions occupy the out-of-plane 3*z* ^2^ *–r* ^2^ orbits [32]. This variation of the orbital occupation in the LSMO/LNO bilayer will increase the localized magnetic moments at the interfacial Mn and Ni ions, which enlarges the interfacial coupling strength eventually. The nearest neighboring spin moment of the ferromagnet in the LSMO layer will always be influenced by an opposing pinning force from the localized magnetic regions, which possibly establishes at the interfacial coupling to allow a spin glass state. The saturation magnetization of the bilayer is smaller than the LSMO single layer due to the fact that interfacial Mn ions are localized, which further supports the interfacial coupling that appears in the LSMO/LNO bilayer [29]. Otherwise, when the LNO layer is on the top, the compressive stress on the interfacial LSMO is weak, and the exchange coupling that occurs in the LNO/LSMO bilayer is inconspicuous, as shown in Fig. 2a.

**Fig. 4:**
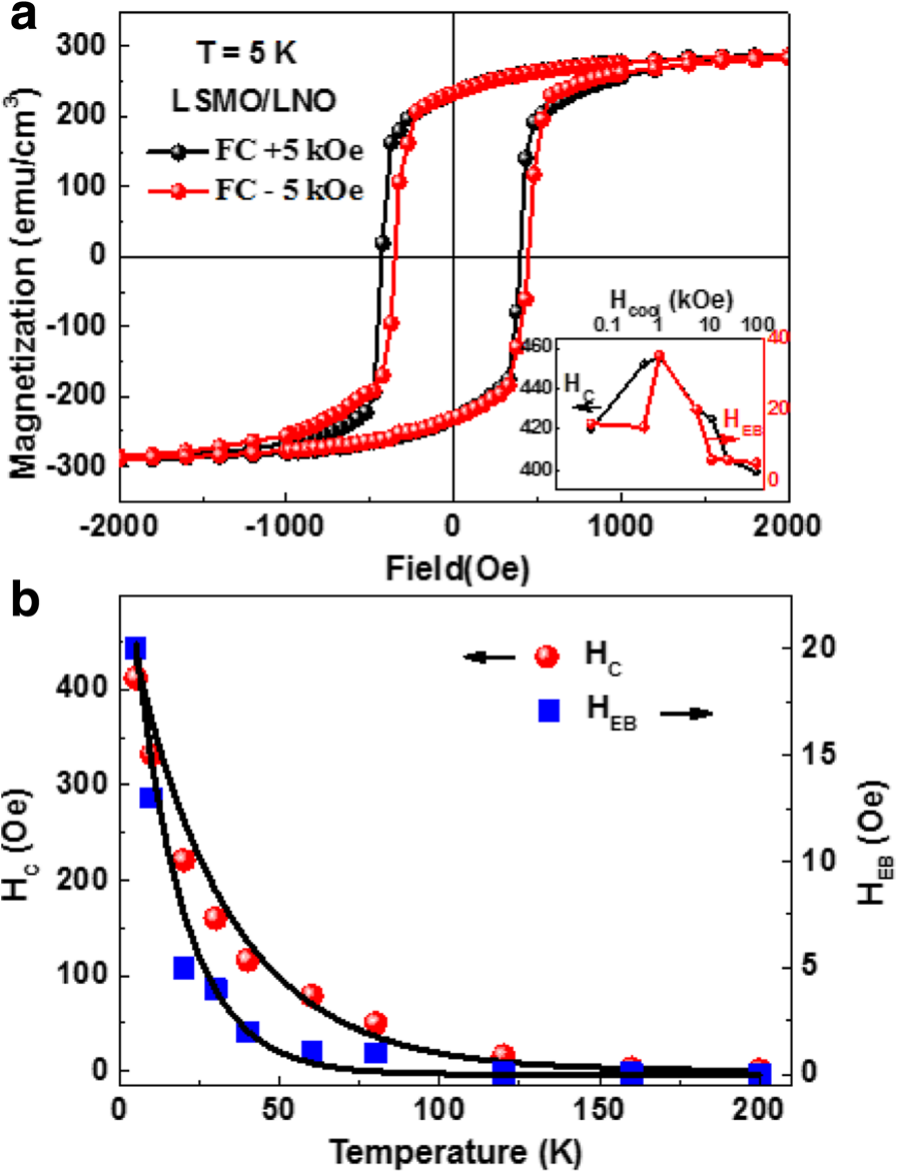
**a** *M*–*H* loops measured on the LSMO/LNO bilayer at 5 K after an in-plain field-cooling (±5 kOe). The *H*_C_ and *H*_EB_ dependence on the cooling field is shown in the inset. **b** Temperature dependence of *H*_EB_ and *H*_C_ for the LSMO/LNO bilayer. The *solid lines* are the fittings to Eq. (3)

The magnetic hysteresis loops of the LSMO/LNO bilayer measured at 5 K after ±5 kOe field cooling from room temperature are shown in Fig. 4a. The hysteresis loops shift along the magnetic field axis, indicating there is an exchange bias effect. After FC in a field of +5 kOe, the hysteresis loop is shifted to the negative field direction and the left and right coercive field is −432 and 392 Oe, respectively. The exchange bias field is 20 Oe. In contrast, the loop is biased in the positive direction with the negative cooling field. The inset of Fig. 4a summarizes the *H*_EB_ and *H*_C_ dependent on different cooling fields. It can be seen that the *H*_EB_ increases rapidly to 35 Oe as the cooling field increases to 1 kOe, and then decreases monotonically to 5 Oe as the cooling field reaches to 70 kOe. The *H*_C_ has a similar trend. It should be noted that there is competition between the spin glass order and the Zeeman coupling, and, in fact, a strong enough magnetic field can destroy the spin glass state entirely [27]. The temperature dependences of *H*_EB_ and *H*_C_ for the LSMO/LNO bilayer are shown in Fig. 4b. It is obvious that *H*_EB_ decreases rapidly with increasing temperature, finally vanishes at the blocking temperature (*T*_B_) of 120 K. As *T*_B_ gets smaller than the freezing temperature *T*_F_ of the spin glass, we suggest that the exchange bias effect in the LSMO/LNO interface is supported by the emergence of the spin glass state. As previously reported, in a range of diverse materials such as LSMO/SMO, the existence of the spin glass state is known to lead an exponential temperature dependent decay of *H*_EB_ and *H*_C_ [29, 33–35]. In Fig. 4b, we fit the temperature dependence of *H*_EB_ and *H*_C_ by the phenomenological formula:

3 $$ \begin{array}{l}{H}_{\mathrm{EB}}(T)={H}_{\mathrm{EB}}^0 \exp \left(- T/{T}_1\right),\\ {}\kern0.5em {H}_{\mathrm{C}}(T)={H}_{\mathrm{C}}^0 \exp \left(- T/{T}_2\right),\end{array} $$

where ${H}_{\mathrm{EB}}^0$ and ${H}_C^0$ are the extrapolations of temperature at zero temperature and *T*_1_ and *T*_2_ are constant. The perfect fitting results give further support that exchange bias in the LSMO/LNO bilayer is controlled by the spin glass state.

## Conclusions

In summary, the interfacial coupling strength can be influenced by the deposition sequence of the bilayer. The coercivity of the bilayer with LNO at the bottom is much higher than that with LNO on the top, indicating that there is a stronger coupling between the interfacial LSMO/LNO bilayer. This strong coupling is due to the presence of a spin glass state in LSMO/LNO bilayer, which is supported by the field dependence of the irreversibility and the magnetic relaxation. Moreover, the temperature and cool field dependence of the exchange bias is attributed to the competition between the spin glass state, thermal disorder, and Zeeman coupling. The interface engineering of the PM/FM heterostructure is promising for inducing many novel physical phenomena.
